# Online learning experiences of secondary school students during COVID-19 – Dataset from Vietnam

**DOI:** 10.1016/j.dib.2022.108662

**Published:** 2022-10-09

**Authors:** Dien Thi Bui, Thuy Thi Nhan, Hue Thi Thu Dang, Trang Thi Thu Phung

**Affiliations:** a The Vietnam National Institute of Educational Sciences, 101 Tran Hung Dao Street, Hoan Kiem District, Hanoi 100000, Vietnam; b Danang Architecture University, 566 Nui Thanh Street, Da Nang 50000, Vietnam

**Keywords:** Online education, Responsive education, COVID-19 education, Online learning platforms, School closure, Student perceptions

## Abstract

This dataset provides an insight into the reality and experiences of online learning as perceived by secondary school students in Vietnam during COVID-related school closures. The dataset addresses four main aspects of online learning, namely (a) students’ access to learning devices, (b) their digital skill readiness, (c) their experience with online learning and assessment activities, and (d) their overall evaluation of the effectiveness of online learning. The survey was administered online via Google Form from September to December 2021 with responses received from 5,327 secondary school students in 5 provinces of Vietnam. The dataset is expected to benefit local educators, administrators, and teachers who are interested in COVID educational practices and pedagogical interventions. The dataset can also benefit international researchers who wish to conduct comparative studies on student online learning or who wish to seek further insight into the responsiveness of an educational system to pandemic situations.

**Specifications Table**

**Table:** 

Subject	Social sciences
Specific subject area	Education Online learning Education during COVID-19
Type of data	Tables Figures Excel file Sav file
How the data were acquired	The data was collected using a Google Forms online survey. The survey link was distributed to students via their class teachers. Student responses were imported into an Excel spreadsheet and analysed using SPSS Version 25.
Data format	Raw Analysed
Description of data collection	The cluster sampling method was used to collect the data. Participating schools were located in 5 provinces, namely Hanoi, Nam Dinh, Quang Binh, Daklak and Can Tho. Targeted respondents for the survey were Grade 6-to-Grade 9 students from 50 secondary schools. A total of 5,327 valid responses were received.
Data source location	Institution: The Vietnam National Institute of Educational Sciences City/Town/Region: Hanoi, Nam Dinh, Quang Binh, Dak Lak and Can Tho Country: Vietnam Latitude and longitude (and GPS coordinates, if possible) for collected samples/data: Hanoi: 21°1′28.2″N, 105°50′28.21″E Nam Dinh: 20° 16′ 45.048″ N 106° 12′ 18.533″ E Quang Binh: 17° 27′ 57.38″ N 106° 35′ 54.226″ E Daklak: 12° 42′ 36.043″ N 108° 14′ 15.907″ E Can Tho: 10°2′13.6″N, 105°47′17.7″E
Data accessibility	Repository name: Mendeley Data Data identification number: 10.17632/cn7vtxdm97.1 Direct URL to data: https://data.mendeley.com/datasets/cn7vtxdm97/1

## Value of the Data

The dataset is expected to have methodological and practical contributions to the topic of online learning.

• In practical terms, the dataset provides a large-scale database of online learning experiences of secondary school students in Vietnam. This can inform Vietnamese educators, administrators, and teachers of the reality and effectiveness of online learning from students’ perspectives, which then can inform the development of action plans, pedagogies, adjustments, or interventions to best support online teaching and learning.
• In methodological terms, the dataset provides a survey tool that local educators and researchers can use to evaluate the effectiveness of online learning or seek means to enhance students' online learning experience. The survey tool in particular and the dataset, in general, can benefit international educators and researchers interested in online education and in the responsiveness of an educational system, particularly in relation to the context of COVID-19 or similar pandemic situations.

## 1 Data Description

The dataset uploaded and referenced at Mendeley data [5] informs the online learning reality of secondary school students in Vietnam during school closures due to COVID-19. It comprises a student questionnaire with 64 items and a raw datafile with 5,327 responses. The questionnaire is structured into four groups, namely (a) demographic information of participating students (3 items), (b) their online learning conditions (16 items), (c) their experience with online learning and assessment activities (37 items), and (d) their overall perception of the effectiveness of online learning (8 items). Demographic items were in the form of selected responses and the remaining items were in the form of 5-point Likert statements.

The first group of information collected was concerned with students’ gender, school grade, and location of residence (Table 1). This demographic information was used to explore correlation with other items in the questionnaire.

**Table 1:** Distribution of student participants by gender, grade, and location.

	Gender		Grade				Location type*		
Total 5327	Male	Female	Grade 6	Grage 7	Grade 8	Grade 9	Urban areas	Rural areas	Mountainous areas
	2429	2898	1069	1563	1398	1297	2234	2675	418

Note.

⁎ Location types are defined in the Vietnamese Government's Decision No. 1211/2016/UBTCQH13 and Decision No. 33/2-2-QĐ-TTg based on localities’ population size and economic indicators. Mountainous areas refer to localities with significant socio-economic disadvantages, with at least 15% of the population belonging to ethnic minority groups and at least 10% of the households living under the national poverty line.

**Fig. 1:**
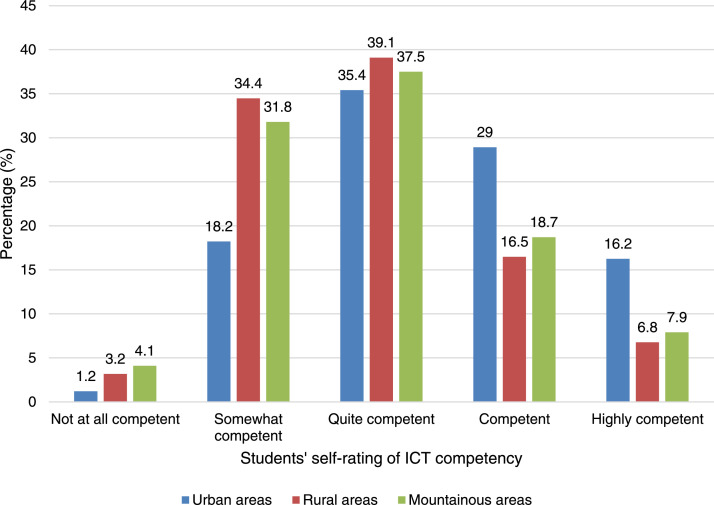
Students’ self-rated ICT skills by location type.

The second group of information was concerned with students’ conditions for online learning. Students were asked whether or not they had access to learning devices, such as tablets, smartphones, or computers connected to the Internet. They were also asked to self-assess their ICT skills, for example, their ability to use online learning platforms and apps to participate in online activities. The data collected was presented in Fig. 1 and in Table 2, Table 3, Table 4. Table 5 presents the data on barriers to students’ online learning.

**Table 2:** Students’ access to learning devices by location type.

	Urban areas		Rural areas		Mountainous areas	
	N	%	N	%	N	%
An Ipad/Tablet with Internet connection	772	34.6	378	14.1	64	15.3
A smart TV with Internet connection	329	14.7	543	20.3	88	21.1
A smartphone with Internet connection	1650	73.9	2390	89.3	356	85.2
A laptop with Internet connection	1318	59.0	716	26.8	164	39.2
A PC (with camera and microphone) with Internet connection	505	22.6	326	12.2	60	14.4
A PC (without camera and microphone) with Internet connection	255	11.4	259	9.7	62	14.8

**Table 3:** Statistical differences in self-rated IT skills of male and female students.

Group Statistics					
	Gender	N	Mean	Std. Deviation	Std. Error Mean
Self-rated IT skills	Male	2429	3.19	1.028	.021
	Female	2898	3.05	.982	.018


Independent Samples Test										
		Levene's Test for Equality of Variances		t-test for Equality of Means						
		F	Sig.	t	df	Sig. (2-tailed)	Mean Difference	Std. Error Difference	95% Confidence Interval of the Difference	
									Lower	Upper
Self-rated IT skills	Equal variances assumed	24.537	.000	5.098	5325	.000	.141	.028	.087	.195
	Equal variances not assumed			5.077	5074.987	.000	.141	.028	.086	.195

**Table 4:** Students’ proficiency in online learning platforms and applications.

	Novice		Advanced Beginner		Intermediate		Proficient		Expert	
	N	%	N	%	N	%	N	%	N	%
Navigating through online learning platforms (e.g., Zoom, Google Meet, Microsoft Teams, etc.)	272	5.1	211	4.0	1321	24.8	2672	50.1	851	16
Using learning platforms or software (e.g., Shub, Kahoot, Menti, etc.) to complete assigned activities	328	6.1	914	17.2	1519	28.5	2019	37.9	547	10.3
Using social networking sites and applications to communicate and interact with teachers and peers	304	5.7	197	3.7	1061	19.9	2684	50.4	1081	20.3

**Table 5:** Barriers to online learning by location type.

	M (Mean ratings on a 5-point Likert scale)		
	Urban areas	Rural areas	Mountainous areas
Poor Internet connection	2.23	2.19	2.29
Lack of online learning facilities	1.55	1.87	2.01
Lack of ICT skills	1.57	2.00	2.07
Lack of (teacher/ school/ parent) support	1.56	1.94	1.96
Health issues	1.42	1.51	1.56
Psychological issues	1.60	1.56	1.66

The third group of information was concerned with students’ online learning experiences, such as their participation in class activities, their interaction with teachers and peers, their experience with assessments, and the forms of teacher support they received. The fourth group of information informed students’ overall perception of the effectiveness of online learning. Statistical analyses showed strong positive correlations between students’ online learning experiences, the level of teacher support, and students’ overall satisfaction of online learning (Table 6).

**Table 6:** Correlations between students' online learning experiences, the level of teacher support, and students' overall satisfaction of online learning.

		Self-rating of IT skills	Barriers to online learning	Online learning experiences	Teacher support for online learning	Overall perception of the effectiveness of online learning
Self-rating of IT skills	Pearson Correlation	1	-.253⁎⁎	.151⁎⁎	.135⁎⁎	.154⁎⁎
	Sig. (2-tailed)		.000	.000	.000	.000
	N	5327	5327	5327	5327	5327

Barriers to online learning	Pearson Correlation	-.253⁎⁎	1	-.098⁎⁎	-.099⁎⁎	-.117⁎⁎
	Sig. (2-tailed)	.000		.000	.000	.000
	N	5327	5327	5327	5327	5327

Online learning experiences	Pearson Correlation	.151⁎⁎	-.098⁎⁎	1	.826⁎⁎	.788⁎⁎
	Sig. (2-tailed)	.000	.000		.000	.000
	N	5327	5327	5327	5327	5327

Teacher support for online learning	Pearson Correlation	.135⁎⁎	-.099⁎⁎	.826⁎⁎	1	.787⁎⁎
	Sig. (2-tailed)	.000	.000	.000		.000
	N	5327	5327	5327	5327	5327

Overall perception of the effectiveness of online learning	Pearson Correlation	.154⁎⁎	-.117⁎⁎	.788⁎⁎	.787⁎⁎	1
	Sig. (2-tailed)	.000	.000	.000	.000	
	N	5327	5327	5327	5327	5327

⁎⁎ Correlation is significant at the 0.01 level (2-tailed).

Statistical differences were found in the level of student participation, teacher support, and overall satisfaction for students from different grades. In particular, Tables 7 and 8 show that students in junior grades (Grade 6 and Grade 7) were more engaged in online learning activities and received more teacher support than those in senior grades (Grade 8 and Grade 9). In the same manner, Table 9 shows that junior students rated more positively their overall experience with online learning than senior peers.

**Table 7:** Online learning experiences of students by grade.

Descriptives								
					95% Confidence Interval for Mean			
	N	Mean	Std. Deviation	Std. Error	Lower Bound	Upper Bound	Minimum	Maximum
Grade 6	1069	3.8722	.82493	.02523	3.8227	3.9217	1	5
Grade 7	1563	3.8489	.84363	.02134	3.8071	3.8908	1	5
Grade 8	1398	3.7964	.82884	.02217	3.7530	3.8399	1	5
Grade 9	1297	3.7683	.74387	.02066	3.7278	3.8088	1	5
Total	5327	3.8202	.81340	.01114	3.7983	3.8420	1	5


Test of Homogeneity of Variances					
		Levene Statistic	df1	df2	Sig.
Online learning experiences	Based on Mean	2.715	3	5323	.043


ANOVA					
	Sum of Squares	df	Mean Square	F	Sig.
Between Groups	8.458	3	2.819	4.269	.005
Within Groups	3515.308	5323	.660		
Total	3523.766	5326			


Robust Tests of Equality of Means				
	Statistic^a^	df1	df2	Sig.
Welch	4.477	3	2863.933	.004


a. Asymptotically F distributed.

**Table 8:** Level of teacher support for online learning by grade.

Descriptives								
					95% Confidence Interval for Mean			
	N	Mean	Std. Deviation	Std. Error	Lower Bound	Upper Bound	Minimum	Maximum
Grade 6	1069	3.7825	.81342	.02488	3.7337	3.8313	1	5
Grade 7	1563	3.7910	.82684	.02091	3.7500	3.8320	1	5
Grade 8	1398	3.7400	.80596	.02156	3.6978	3.7823	1	5
Grade 9	1297	3.6960	.75069	.02084	3.6551	3.7369	1	5
Total	5327	3.7528	.80133	.01098	3.7313	3.7743	1	5


Test of Homogeneity of Variances					
		Levene Statistic	df1	df2	Sig.
Teacher support for online learning	Based on Mean	1.809	3	5323	.143


ANOVA					
	Sum of Squares	df	Mean Square	F	Sig.
Between Groups	7.632	3	2.544	3.968	.008
Within Groups	3412.320	5323	.641		
Total	3419.951	5326			


Robust Tests of Equality of Means				
	Statistic^a^	df1	df2	Sig.
Welch	4.154	3	2862.605	.006

a. Asymptotically F distributed.

**Table 9:** Students’ overall perception of the effectiveness of online learning by grade.

Descriptives								
					95% Confidence Interval for Mean			
	N	Mean	Std. Deviation	Std. Error	Lower Bound	Upper Bound	Minimum	Maximum
Grade 6	1069	3.8182	.78447	.02399	3.7711	3.8653	1	5
Grade 7	1563	3.7935	.81971	.02073	3.7528	3.8342	1	5
Grade 8	1398	3.6999	.81613	.02183	3.6571	3.7427	1	5
Grade 9	1297	3.6347	.74045	.02056	3.5944	3.6751	1	5
Total	5327	3.7352	.79608	.01091	3.7139	3.7566	1	5


Test of Homogeneity of Variances					
		Levene Statistic	df1	df2	Sig.
Overall perception of the effectiveness of online learning	Based on Mean	1.307	3	5323	.270


ANOVA					
	Sum of Squares	df	Mean Square	F	Sig.
Between Groups	27.503	3	9.168	14.577	.000
Within Groups	3347.821	5323	.629		
Total	3375.324	5326			


Robust Tests of Equality of Means				
	Statistic^a^	df1	df2	Sig.
Welch	15.405	3	2870.246	.000

a. Asymptotically F distributed.

When location types were factored in, statistical differences were also found in the level of teacher support, student participation, and students’ overall satisfaction with online learning, as shown in Table 10, Table 11, Table 12.

**Table 10:** Online learning experiences of students by location type.

Descriptives								
Online learning experiences								
					95% Confidence Interval for Mean			
	N	Mean	Std. Deviation	Std. Error	Lower Bound	Upper Bound	Minimum	Maximum
Urban	2234	3.8789	.77573	.01641	3.8467	3.9111	1	5
Rural	2675	3.8022	.81920	.01584	3.7712	3.8333	1	5
Mountainous	418	3.6213	.92975	.04548	3.5319	3.7107	1	5
Total	5327	3.8202	.81340	.01114	3.7983	3.8420	1	5


Test of Homogeneity of Variances					
		Levene Statistic	df1	df2	Sig.
Online learning experiences	Based on Mean	11.188	2	5324	.000


ANOVA					
	Sum of Squares	df	Mean Square	F	Sig.
Between Groups	25.088	2	12.544	19.089	.000
Within Groups	3498.678	5324	.657		
Total	3523.766	5326			


Robust Tests of Equality of Means				
	Statistic^a^	df1	df2	Sig.
Welch	16.435	2	1132.344	.000

a. Asymptotically F distributed.

**Table 11:** Level of teacher support for online learning by location type.

Descriptives								
					95% Confidence Interval for Mean			
	N	Mean	Std. Deviation	Std. Error	Lower Bound	Upper Bound	Minimum	Maximum
Urban	2234	3.8133	.77882	.01648	3.7810	3.8456	1	5
Rural	2675	3.7353	.79647	.01540	3.7051	3.7655	1	5
Mountainous	418	3.5415	.90500	.04426	3.4545	3.6285	1	5
Total	5327	3.7528	.80133	.01098	3.7313	3.7743	1	5


Test of Homogeneity of Variances					
		Levene Statistic	df1	df2	Sig.
Teacher support for online learning	Based on Mean	9.723	2	5324	.000


ANOVA					
	Sum of Squares	df	Mean Square	F	Sig.
Between Groups	27.653	2	13.826	21.700	.000
Within Groups	3392.299	5324	.637		
Total	3419.951	5326			


Robust Tests of Equality of Means				
	Statistic^a^	df1	df2	Sig.
Welch	18.661	2	1134.599	.000

a. Asymptotically F distributed.

**Table 12:** Students’ overall perception of the effectiveness of online learning by location type.

Descriptives								
					95% Confidence Interval for Mean			
	N	Mean	Std. Deviation	Std. Error	Lower Bound	Upper Bound	Minimum	Maximum
Urban	2234	3.7405	.79603	.01684	3.7075	3.7735	1	5
Rural	2675	3.7562	.78188	.01512	3.7266	3.7859	1	5
Mountainous	418	3.5730	.86688	.04240	3.4896	3.6563	1	5
Total	5327	3.7352	.79608	.01091	3.7139	3.7566	1	5


Test of Homogeneity of Variances					
		Levene Statistic	df1	df2	Sig.
Overall perception of the effectiveness of online learning	Based on Mean	10.264	2	5324	.000


ANOVA					
	Sum of Squares	df	Mean Square	F	Sig.
Between Groups	12.245	2	6.123	9.693	.000
Within Groups	3363.078	5324	.632		
Total	3375.324	5326			


Robust Tests of Equality of Means				
	Statistic^a^	df1	df2	Sig.
Welch	8.306	2	1143.310	.000

a. Asymptotically F distributed.

## 2 Experimental Design, Materials and Methods

The COVID-19 pandemic has globally affected all aspects of life, including education [10]. Many countries have had to change their education strategies and plans, including shifting from face-to-face learning to online learning to ensure safety for students, educators as well as wider communities [11]. The large-scale, long-term implications of online learning are unprecedented. This highlights the significance of data on online learning to help define appropriate steps to respond to the pandemic and similar situations in the future [2,7].

This dataset was one outcome of a research project conducted to propose an adaptive educational model for schools in the context of a pandemic. The main data collection tool was a questionnaire developed by the research team based on the Online Education Framework and Theories [9] and an extensive review of studies on online education and influencing factors in the context of education in the pandemic (such as [1,^3^,^4^,^6^,^8^,^11^,12]). The questionnaire considered Vietnam's practical school settings and was validated with expert judgements and piloted before being implemented on a large scale. It focused on the practical experience of Vietnamese students in online learning, factors influencing their online learning conditions, and teachers’ pedagogical and assessment modalities used in online teaching strategies. The targeted research participants were school students in Grades 6 to 9 – These grades are the last level of compulsory education in the Vietnamese educational system and serve as an important learning period before students decide to pursue further education or work. To ensure the currency and validity of the data collection tool, the questionnaire was informed by a literature review and consulted with experts. It was then adapted into the format of an online survey with a combination of mandatory and optional questions to be administered on Google Forms. The questionnaire was piloted on 80 students and revised for wording and number of items before being distributed to local Departments of Education and Training to seek approval for being administered on a large scale. The questionnaire has high internal consistency with a Cronbach's alpha value of 0.954.

Five provinces were chosen for the survey, namely Ha Noi, Nam Dinh, Quang Binh, Dak Lak, and Can Tho. These provinces are representative of Northern, Central, and Southern Vietnam and experienced heavy school closure due to the COVID-19 pandemic. 50 public schools from the provinces participated in the survey, representing schools with different closure and online teaching policies and schools from different locality types, namely rural, urban, and mountainous schools. Participation from each school, on average, was about 100 students in Grade 6 to Grade 9. The project information and consent forms were sent to students’ parents via class teachers. After parents’ consent was received, the online link to the survey was distributed directly to students. Reminders were sent one week later via class teachers who acted as a communication channel between the research team, students, and parents. A total of 6,380 responses were collected, 1,053 of which were removed due to systemic missing data. The response rate was 83.4%, which was a good response rate considering the survey was conducted online and on a voluntary basis. 5,327 responses were analysed using IBM SPSS Version 25.

## Ethics Statements

The procedure for conducting this research was approved and monitored by the Ethics Committee of the Vietnam National Institute of Educational Sciences (the ethics approval number- B2021-VKG-01.GRANTED).The procedure for collecting data strictly adhered to the ethical guidelines and regulations of the committee in charge. Students, class teachers, and parents were informed of the research and provided parents’ consent before students’ responses were collected.

## CRediT authorship contribution statement

**Dien Thi Bui:** Methodology, Writing – original draft, Supervision. **Thuy Thi Nhan:** Methodology, Writing – review & editing. **Hue Thi Thu Dang:** Methodology, Writing – review & editing. **Trang Thi Thu Phung:** Software, Data curation, Validation.

## Declaration of Competing Interest

The authors declare that they have no known competing financial interests or personal relationships that could have appeared to influence the work reported in this paper.

## Data Availability

Online learning during COVID-19 Pandemic- Dataset from Vietnam (Original data) (Mendeley Data).
